# Efficient Red Thermally Activated Delayed Fluorescence Emitters Based on a Dibenzonitrile-Substituted Dipyrido[3,2-a:2′,3′-c]phenazine Acceptor

**DOI:** 10.3390/molecules26092427

**Published:** 2021-04-22

**Authors:** Lin He, Xuan Zeng, Weimin Ning, Ao Ying, Yunbai Luo, Shaolong Gong

**Affiliations:** Department of Chemistry, Hubei Key Laboratory on Organic and Polymeric Optoelectronic Materials, Wuhan University, Wuhan 430072, China; lin_he@whu.edu.cn (L.H.); zengx@whu.edu.cn (X.Z.); wmning@whu.edu.cn (W.N.); yingao96@whu.edu.cn (A.Y.)

**Keywords:** organic light-emitting diodes, thermally activated delay fluorescence, red emitter, electron acceptor

## Abstract

How to construct efficient red-emitting thermally activated delayed fluorescence (TADF) materials is a challenging task in the field of organic light-emitting diodes (OLEDs). Herein, an electron acceptor moiety, 3,6-DCNB-DPPZ, with high rigidity and strong acceptor strength was designed by introducing two cyanobenzene groups into the 3,6-positions of a dipyrido[3,2-a:2′,3′-c]phenazine unit. A red-emitting compound, 3,6_R, has been designed and synthesized by combining the rigid acceptor unit with two triphenylamine donors. Due to high molecular rigidity and strong intramolecular charge transfer characteristic in donor–acceptor–donor skeleton, 3,6_R exhibited a red emission with a high photoluminescence quantum yield of 86% and distinct TADF nature with short delayed fluorescence lifetime of about 1 microsecond. Accordingly, the OLED using 3,6_R as the guest emitter gained a high external quantum efficiency of 12.0% in the red region with an electroluminescence peak of 619 nm and favorable Commission Internationale de l’Eclairage coordinates of (0.62, 0.38).

## 1. Introduction

During the past decade, purely organic emitters exhibiting thermally activated delay fluorescence (TADF) have garnered considerable attention in the field of organic light-emitting diodes (OLEDs) because of their superior ability of harvesting both singlet and triplet excitons for luminance without the use of noble metals [1,2,3,4,5]. By accessing an effective reverse intersystem crossing (RISC) process from the lowest triplet to singlet state with a small singlet-triplet energy gap (Δ*E*_ST_), TADF emitters can theoretically achieve up to 100% internal quantum efficiency in OLEDs [6,7,8]. Nowadays, efficient organic TADF emitters have been reported in the blue [9,10,11] and green [12,13,14] region, facilitated by rational molecular design. Despite this achievement, efficient red-emitting TADF emitters are difficult to construct, especially in the color region having Commission Internationale de l’Eclairage (CIE) coordinates of (x > 0.60, y < 0.40) [15,16,17,18].

Generally, a small ΔE_ST_ to enable an effective RISC process and a strong charge transfer (CT) state to afford a red emission color are two basic design principles for efficient red-emitting TADF emitters [19]. These requirements are inherently detrimental to photoluminescence quantum yield (Φ_PL_) of red emitters because the spatially isolated frontier molecular orbital distribution required for the small Δ*E*_ST_ normally degrades radiative rate of S_1_ and the narrow energy gaps generally lead to severe non-radiative processes from S_1_ to the ground state [16,17,18]. Accordingly, red TADF emitters often face the primary limitation of low Φ_PL_ [19,20,21]. To address this issue, electron donor (D) and/or acceptor (A) moieties with high rigidity are usually adopted in the pre-twisted D-A or D-A-D motif for designing red TADF emitters [22,23,24,25,26]. So far, a few electron acceptors with strong acceptor strength and large conjugation have been reported to construct red TADF emitters, for example, heptazine [27], dibenzo[f,h]quinoxaline-2,3-dicarbonitrile [26,28], and dibenzo[a,c]phenazine-3,6-dicarbonitrile [29,30]. Despite these progresses, most of red TADF emitters have moderate Φ_PL_ values of below 70% [19,31]. Thus, further efforts are still required to design new electron acceptors with high rigidity and strong acceptor strength for constructing red TADF emitters with satisfactory CIE coordinates and high Φ_PL_s.

In this study, we integrated two cyanobenzene groups into the dipyrido[3,2-a:2′,3′-c]phenazine core to construct a new electron acceptor moiety, 3,6-DCNB-DPPZ. Combining with two triphenylamine donors in the D-A-D motif, we designed and synthesized a red TADF molecule, 4,4′-(11,12-bis(4-(diphenylamino)phenyl)dipyrido[3,2-a:2′,3′-c]-phenazine-3,6-diyl)dibenzonitrile (3,6_R). Benefiting from the high-rigidity molecular skeleton and strong CT transition between the electron donor and accept-or unit, 3,6_R exhibited a satisfactory red emission peaking at above 610 nm, together with a high Φ_PL_ of 86% and the impressive external quantum efficiency of 12%.

## 2. Results and Discussion

**Scheme 1:**
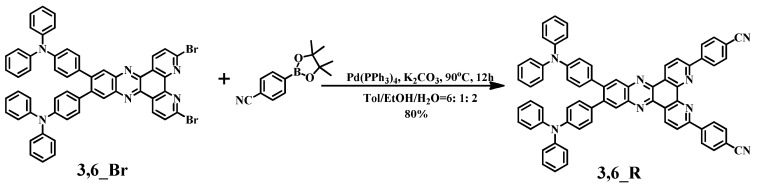
Synthetic route of 3,6_R.

As depicted in Scheme 1, the target compound of 3,6_R was synthesized in two steps. The key intermediate of 3,6_Br was synthesized via a dehydration cyclization reaction using *N^4^*,*N^4^*,*N^4^*′′,*N^4^*′′-tetraphenyl-[1,1′:2′,1′′-terphenyl]-4,4′,4′′,5′-tetraamine and 2,9-dibromo-4a,6a-dihydro-1,10-phenanthroline-5,6-dione (Scheme S1). Afterward, the target compound 3,6_R was synthesized by a palladium catalyzed Suzuki-Miyaura cross-coupling reaction of 3,6_Br with 4-(4,4,5,5-tetramethyl-1,3,2-dioxaborolan-2-yl)benzonitrile in a good yield (Scheme 1). The key intermediate and target compound were characterized and confirmed via ^1^H-NMR spectroscopy (Figures S3 and S4 in Supplementary Materials)*,* high-resolution mass spectrometry (HRMS) (Figure S5)*,* and elemental analysis.

**Figure 1:**
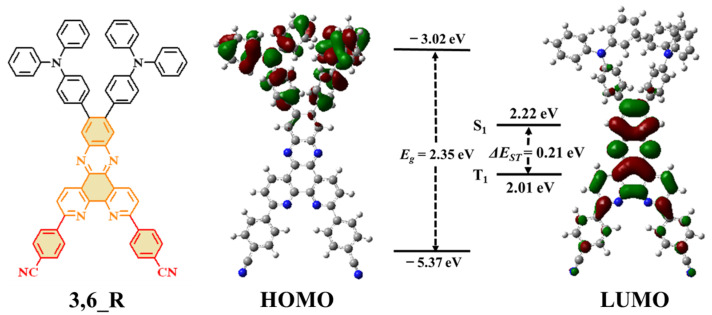
Molecular structure, frontier molecular orbital distributions and energy levels of lowest-lying singlet and triplet excited states of 3,6_R obtained by theoretical calculations.

Theoretical calculations based on density functional theory (DFT) and time-dependent DFT (TD-DFT) were carried out on the molecule to predict its molecular characteristics and energy levels. The optimized ground state structure (Figure S1) revealed that 3,6_R had relatively high dihedral angles of 50–63° between the TPA donor and the 3,6-DCNB-DPPZ acceptor core. This guarantees well-separated frontier molecular orbital distributions for the molecule (Figure 1), together with the HOMO and LUMO mainly located on the TPA donor and the 3,6-DCNB-DPPZ acceptor core, respectively. Furthermore, 3,6_R had a small torsion angle of 10° between the cyanobenzene acceptor subunit and the DPPZ core. Such planar acceptor structure of 3,6-DCNB-DPPZ leads to some degree of LUMO distribution on the cyanobenzene acceptor subunit for 3,6_R. Accordingly, the delocalized conjugation in the 3,6-DCNB-DPPZ acceptor made 3,6_R have a deepened LUMO level of −3.02 eV. This result suggests that the 3,6-DCNB-DPPZ acceptor has strong acceptor strength. 3,6_R had relatively low S_1_/T_1_ energies of 2.22/2.01 eV, which can be attributed to the strong CT transition from the TPA donor to the 3,6-DCNB-DPPZ acceptor. Accordingly, 3,6_R had a small ΔE_ST_ value of 0.21 eV, favorable to access a TADF channel. The natural transition orbitals (NTOs) (Figure S2) on excited states revealed that the S_1_ of 3,6_R had intramolecular CT character; while its T_1_ possessed CT/locally excited (LE) hybrid NTO distributions. The different NTO natures of S_1_ and T_1_ may benefit up-conversion process from T_1_ to S_1_ according to the El-Sayed rule [32].

**Figure 2:**
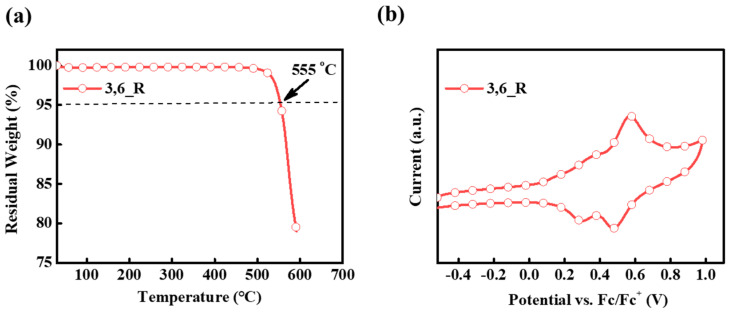
(**a**) TGA curve of 3,6_R. (**b**) Oxidation behavior of 3,6_R.

The thermal stability of the compound was investigated by thermo-gravimetric analysis (TGA) and differential scanning calorimetry (DSC) measurements. As shown in Figure 2a, the decomposition temperature (T_d_ with a 5 wt% loss) of 3,6_R was determined as high as 555 °C. Apparently, the planar and rigid structure endows 3,6_R with pretty good thermal stability. Furthermore, no clear glass transition temperature was observed in the DSC curve, which could be associated with high molecular rigidity of the compound. These results implied that the compound had excellent thermal stability and thus could be suitable for vacuum deposition.

The electrochemical properties of the compound were tested by cyclic voltammetry using ferrocenium/ferrocene as an internal reference. As shown in Figure 2b, the compound experienced a quasi-reversible oxidation process at + 0.55 V (calibrated versus ferrocenium/ferrocene) assigned to the triphenylamine donor. Furthermore, the HOMO energy level of 3,6_R was determined to be −5.32 eV, according to half-wave potential. As calculated from the difference between the HOMO level and the optical band gap (E_g_), the LUMO energy level of 3,6_R was calculated to be −3.06 eV. These results are basically consistent with the abovementioned theoretical results.

**Figure 3:**
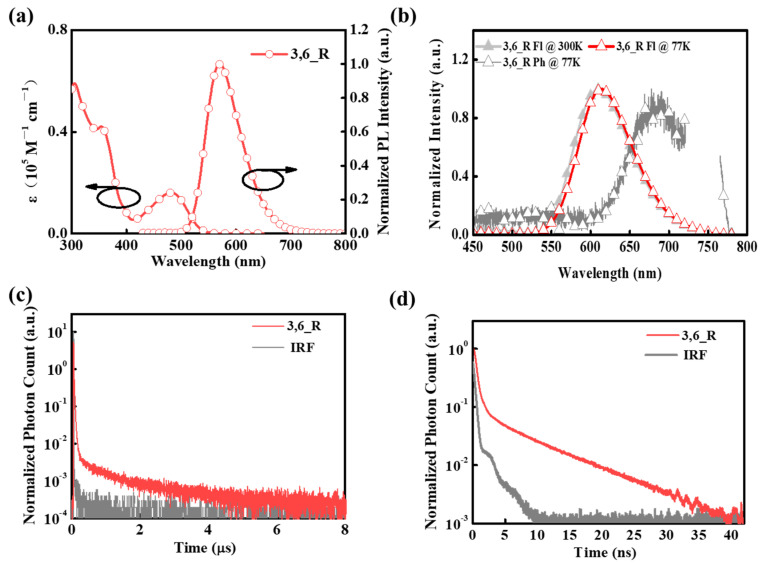
(**a**) UV-Vis absorption and normalized PL spectra of 3,6_R in 10^−5^ M toluene solution at 300 K. (**b**) Normalized fluorescence (measured at 300 and 77 K) and phosphorescence spectra (measured at 77 K) of 10 wt% 3,6_R doped into the DPEPO host. (**c**) Transient photoluminescence decay curves of 10 wt% 3,6_R doped into the DPEPO host. (**d**) The prompt fluorescence decay of 10 wt% 3,6_R doped into the DPEPO film.

The UV-Vis absorption and fluorescence spectra of 3,6_R in 10^−5^ M toluene was shown in Figure 3a. 3,6_R had a strong absorption band at 355 nm (Table 1), which can be assigned to π-π* transition of the LE state of DPPZ-BCN moiety. Weak and broad absorption band at 480 nm for the compound was recognized as typical CT transition from the TPA donor to the 3,6-DCNB-DPPZ acceptor. Moreover, 3,6_R displayed a strong and structureless orange-red emission with a main emission peak at 571 nm in dilute toluene solution. Furthermore, 3,6_R exhibited a significant positive solvatochromic effect with a remarkable redshift of 90 nm when varying the solvent from low-polarity toluene to high-polarity dichloromethane (Figure S6). This strongly suggests obvious CT characteristic of S_1_ for the emitter. Subsequently, the emissive characteristics of the emitter in the film state was also studied by employing a widely used phosphine oxide host matrix of bis(2-(diphenylphosphino)phenyl)ether oxide (DPEPO). 3,6_R in the DPEPO host displayed CT-featured emission profile with a main emission peak at 613 nm at room temperature. Moreover, its phosphorescence spectrum in the DPEPO films at a low temperature of 77 K was dominant by structureless and broad emission profile derived from the typical CT transition of T_1_. On the basis of onset wavelengths of fluorescence and phosphorescence spectra, 3,6_R had low S_1_/T_1_ levels of 2.28/2.06 eV, respectively, which can be rationalized by the strong CT transition from the TPA donor to the 3,6-DCNB-DPPZ acceptor. Accordingly, 3,6_R in the DPEPO host had a small ΔE_ST_ value of 0.22 eV, in good consistence with the theoretical results. The small ΔE_ST_ value could access the TADF process for the emitter in the film state.

To confirm TADF character, we further measured the transient PL decay curves of 3,6_R in the DPEPO host. As shown in Figure 3c,d, the transient PL decays of 3,6_R experienced double exponential decays composed of prompt and delayed fluorescence components together with the fitting lifetimes of 20 ns and 1.04 μs. Such a short delayed fluorescence lifetime of ~1 μs manifested that efficient RISC from T_1_ to S_1_ occurred in the emitter and thus efficient utilization of triplet excitons could be expected in photo- and electroluminescence process. Thanks to high rigidity in the molecular skeleton, 3,6_R in the DPEPO host had a high Φ_PL_ of 86%. To better understand the emissive processes of the emitter, the corresponding rate constant of radiative transition (*k_r, S_*) and RISC (*k_RISC_*) were calculated using related equations (Equations S1–6 in the supporting information). 3,6_R had a moderate *k*_RISC_ of 6.7 × 10^5^ s^−1^ (Table 1 and Table S2), on par with typical TADF emitters. Moreover, the emitter possessed high *k_r,S_* of 3.6 × 10^7^ s^−1^, 6.1-fold larger than its corresponding nonradiative rate constant. Such a high rate constant of radiative transition and RISC process implied that 3,6_R can efficiently harvest most excitons in the emissive process. This may be beneficial to obtain good device performance in the OLEDs.

**Figure 4:**
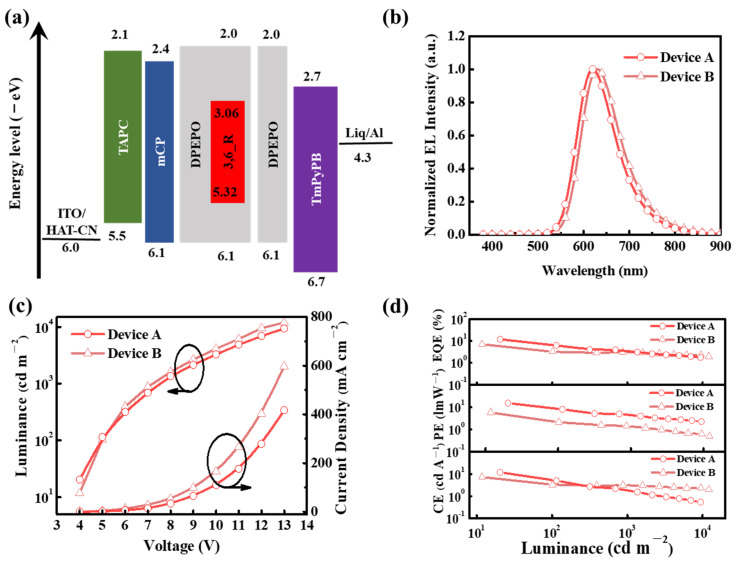
(**a**) The energy level diagrams of devices A and B. (**b**) Electroluminescence spectra of devices A and B. (**c**) Luminance–voltage–current density curves of devices A and B. (**d**) External quantum efficiency, power efficiency, and current efficiency as a function of luminance for devices A and B.

To evaluate electroluminescence properties of 3,6_R, the doped devices with a typical configuration (Figure 4a and Figure S8) were fabricated. It was ITO/HAT-CN (5 nm)/TAPC (30 nm)/mCP (10 nm)/DPEPO: 3,6_R (10 or 30 wt%, 20 nm)/DPEPO (10 nm)/TmPyPB (30 nm)/Liq (1.5 nm)/Al (100 nm). ITO (indium tin oxide) and Al acted as anode and cathode, respectively. HAT-CN (1,4,5,8,9,11-hexaazatriphenylene-hexanitrile) and Liq (8-hydroxyquinolinato lithium) were used as the hole- and electron-injecting layer, respectively. TAPC (1,1-bis[(di-4-tolylamino)phenyl]cyclohexane) and TmPyPB (1,3,5-tri(m-pyrid-3-yl-phenyl)benzene) functioned as hole- and electron-transporting layer, respectively. mCP (1,3-carbazolbenze-ne) and DPEPO acted as exciton-blocking layers, respectively; 3,6_R was doped into the DPEPO host material with the doping concentrations of 10 wt% (device A) and 30 wt% (device B) to serve as the emissive layers, respectively. The energy level diagram and corresponding molecular structures of devices A and B were shown in Figure 4a and Figure S8.

Electroluminescence (EL) characteristics of the 3,6_R-based OLEDs are shown in Figure 4b–d and the key EL data were summarized in Table 2. Similar to the PL spectra in the DPEPO host, device A displayed a red EL profile peaking at 619 nm, together with favorable CIE coordinates of (0.62, 0.38). With the increasing doping concentration from 10 to 30 wt%, the EL peak wavelength red-shifted to 629 nm for device B, corresponding to CIE coordinates of (0.64, 0.36). Meanwhile, no detectable host emission peaking at the range of 370–450 nm was observed for both devices, suggesting excellent exciton confinement in the emitter 3,6_R. Both devices exhibited similar current density–voltage-luminance characteristics with the same turn-on voltages of ~4.0 V. Thanks to the high PLQY of 86%, 3,6_R supported its devices with outstanding EL performance. The optimal device A delivered a maximum external quantum efficiency of 12.0%, a maximum current efficiency of 15.3 cd A^−1^, and a maximum power efficiency of 12.0 lm W^−1^. The non-ideal device performance could be associated with the relatively weak triplet harvesting ability of 3,6_R in the EL process originating from its low delayed fluorescence ratio of 14% and moderate *k*_RISC_.

## 3. Materials and Methods

Synthesis of 4,4′-(11,12-bis(4-(diphenylamino)phenyl)dipyrido[3,2-a:2′,3′-c]phena-zine-3,6-diyl)dibenzonitrile (3,6_R)

Pd(PPh_3_)_4_ (20 mg, 0.02 mmol), K_2_CO_3_ (829 mg, 6.00 mmol), 4-(4,4,5,5-tetramethyl-1,3,2-dioxaborolan-2-yl)benzonitrile (122 mg, 0.54 mmol), 4,4′-(3,6-dibromodipyrido[3,2-a:2′,3′-c]phenazine-11,12-diyl)bis(*N,N*-diphenylaniline) (3,6_Br, 200 mg, 0.22 mmol) in a mixed solvent containing 12 mL toluene, 2 mL ethanol, and 4 mL water were stirred overnight at 90 °C under argon atmosphere. Afterwards, the reaction mixture was poured into 200 mL water, extracted with dichloromethane for three times, dried over with anhydrous Na_2_SO_4_. After the removal of solvents, the residue was purified using column chromatography with dichloromethane/ethyl acetate (4:1 *v*/*v*) as the eluent to give a red powder (168 mg, yield: 80%). ^1^H-NMR (400 MHz, CDCl_3_ + TMS, 25 °C): δ 9.72 (d, *J* = 8.4 Hz, 2H), 8.56 (d, *J* = 8.4 Hz, 4H), 8.36 (s, 2H), 8.29 (d, *J* = 8.2 Hz, 2H), 7.91 (d, *J* = 8.3 Hz, 4H), 7.31–7.28 (m, 8H), 7.22 (d, *J* = 8.5 Hz, 4H), 7.15 (d, *J* = 7.6 Hz, 8H), 7.09–7.04 (m, 8H). Elemental analysis (%) for C_68_H_42_N_8_: C, 84.10; H, 4.36; N, 11.54; found: C, 84.17; H, 4.46; N, 11.67. HRMS (ESI) *m*/*z* calcd for C_68_H_42_N_8_Na^+^ [M + Na]^+^ 993.3424, found 993.3421.

## 4. Conclusions

In summary, we have constructed a new electron acceptor unit of 3,6-DCNB-DPPZ by introducing two cyanobenzene groups into 3,6-positions of dipyrido[3,2-a:2′,3′-c]phenazine unit and further designed and synthesized the red emitter of 3,6_R by combining this rigid electron acceptor with two triphenylamine donors. Thanks to the strong charge transfer transition and high molecular rigidity, 3,6_R had good thermal stability, satisfactory red emission, high photoluminescence quantum yield, and distinct TADF nature with short delayed fluorescence lifetime. Based on these excellent properties, the 3,6_R-based device delivered good electroluminescence performance with the maximum external quantum efficiency of 12.0%, together with an emissive peak at 619 nm and corresponding CIE coordinates of (0.62, 0.38). This finding presents a feasible pathway for constructing efficient red-emitting TADF emitters on the basis of newly designed electron acceptors. Further studies on deep-red/near-infrared TADF emitters bearing new electron acceptors are ongoing.

## Figures and Tables

**Table 1:** Photophysical properties of 3,6_R.

Compound	λ_abs_ [nm] ^a^	λ_em_ [nm] ^b^	τ_p_/τ_d_ [ns]/[μs] ^c^	Φ_p_/Φ_d_ [%] ^d^	Φ_PL_ [%] ^e^	*k*_r,S_/*k*_RISC_ [10^7^ s^−1^] ^f^
3,6_R	355/480	571/613	20/1.04	72/14	86	3.6/0.067

^a^ Measured in 10^−5^ M toluene solution. ^b^ Measured in 10^−5^ M toluene solution and in DPEPO film with 10 wt% doping concentration. ^c^ Measured in 10 wt% DPEPO-doped film. ^d^ The prompt and delayed fluorescence photoluminescence quantum yield of 10 wt% 3,6_R doped into DPEPO film under oxygen-free condition. ^e^ The rate constants of radiative singlet excitons and RISC process of 10 wt% 3,6_R doped into DPEPO film. ^f^ Radiation transition rate constants and reverse intersystem crossing (RISC) rate constants of 10 wt% 3,6_R doped into DPEPO film.

**Table 2:** Electroluminescence characteristics of the devices.

Devices	V_on_ [V] ^a^	λ_EL_ [nm] ^b^	L_max_ [cd m^−2^] ^c^	CE [cd A^−1^] ^d^	PE [lm W^−1^] ^e^	EQE [%] ^f^	CIE (x,y) ^g^
A	4.0	619	9382	15.3/5.5/1.9	12.0/8.7/4.5	12.0/6.6/3.5	(0.62, 0.38)
B	4.0	629	12100	7.4/2.1/1.4	5.8/3.6/3.2	7.1/3.2/3.0	(0.64, 0.36)

^a^ The turn on voltage at the luminance of above 1 cd m^−2^. ^b^ EL peak of the devices. ^c^ Maximum luminance of the devices. ^d^ Current efficiency at maximum, 100, and 1000 cd m^−2^. ^e^ Power efficiency at maximum, 100, and 1000 cd m^−2^. ^f^ External quantum efficiency at maximum, 100, and 1000 cd m^−2^. ^g^ Commission Internationale de l’Eclairage (CIE) coordinates.

## Data Availability

The data presented in this study are available on request from the corresponding author.

## Supplementary Materials

The following are available online: computational methods; details for device fabrications and measurements; key intermediate synthesis route (Scheme S1); optimal geometric construction (Figure S1); NTOs (Figure S2); physical properties and rate constants (Tables S1 and S2); ^1^H-NMR spectroscopy of key intermediate and target compound (Figures S3 and S4); HRMS (ESI) (Figure S5); solvents polarization effect (Figure S6); chemical structures of the materials employed in the vacuum-deposited devices (Figure S7).

## Sample Availability

Samples of the compounds are available from the corresponding author. However, it may be necessary to pay properly for the synthesis and mailing of samples.
