# Supplementary material for: Efficient Red Thermally Activated Delayed Fluorescence Emitters Based on a Dibenzonitrile-Substituted Dipyrido[3,2-a:2′,3′-c]phenazine Acceptor
Source: Molecules. 2021 Apr 22;26(9):2427. doi: 10.3390/molecules26092427 (PMC8122472; doi:10.3390/molecules26092427)
Supplement: Supplementary file 1 [file molecules-26-02427-s001.zip › molecules-1199655-supplementary.pdf]

Supporting Information

Efficient Red Thermally Activated Delayed Fluorescence Emitters based on Dibenzonitrile-Substituted Dipyrido[3,2-a:2',3'-c]phenazine Acceptor

Lin He, Xuan Zeng, Weiming Ning, Ao Ying, Yunbai Luo* and Shaolong Gong*

Department of Chemistry, Hubei Key Laboratory on Organic and Polymeric Optoelectronic Materials, Wuhan University, Wuhan 430072, People's Republic of China. lin_he@whu.edu.cn (L.H.); zengx@whu.edu.cn (X.Z.); wmning@whu.edu.cn (W.N.); yingao96@whu.edu.cn (A.Y.).

*Correspondence: slgong@whu.edu.cn (S.G.); ybai@whu.edu.cn (Y.L.)

General information

All reagents and raw materials are used as received from commercial sources without further purification. *N*^{4},*N*^{4},*N*$^{4''}$,*N*$^{4''}$-tetraphenyl-[1,1':2',1''-terphenyl]-4,4',4'',5'-tetraamine was prepared according to the literature[1]. ^{1}H NMR spectra was measured on a Bruker Advanced II (400 MHz) spectrometers or MERCURYVX300. High-resolution mass spectra (HRMS) were measured on a LCQ-Orbitrap Elite (Thermo-Fisher Scientific, Waltham, MA, USA) mass spectrometer. Thermogravimetric analysis (TGA) was undertaken with a NETZSCHSTA 449C instrument. The thermal stability of the samples under a nitrogen atmosphere was determined by measuring their weight loss while heating at a rate of 10 ºC min^{-1} from 25 to 600 ºC. Cyclic voltammetry (CV) was carried out in nitrogen-purged dichloromethane (DCM) at room temperature with a CHI voltammetric analyser. Tetrabutylammonium hexafluorophosphate ($TBAPF_6$) (0.1 M) was used as the supporting electrolyte. The conventional three-electrode configuration consists of a platinum working electrode, a platinum wire auxiliary electrode, and an Ag wire pseudo-reference electrode with ferrocenium-ferrocene (Fc^+/Fc) as the internal standard. Cyclic voltammograms were obtained at a scan rate of 100 mV s^{-1}. Formal potentials are calculated as the average of cyclic voltammetric anodic and cathodic peaks. The ground state molecular structures were optimized at the B3LYP-D3(BJ)/def2-SVP level of theory. Energy gaps between first singlet state (S_1) and triplet state (T_1) were calculated using the TDDFT based on the PBE0/def2-SVP level of theory on the optimized geometry.

Synthesis of 4,4'-(3,6-dibromodipyrido[3,2-a:2',3'-c]phenazine-11,12-diyl)bis(*N,N*-diphenylaniline) (3,6_Br)

A mixture of $N^4,N^4,N^{4''},N^{4''}$-tetraphenyl-[1,1':2',1''-terphenyl]-4,4',4'',5'-tetraamine (268 mg, 0.45 mmol), 2,9-dibromo-4a,6a-dihydro-1,10-phenanthroline-5,6-dione (149 mg, 0.41 mmol), 5 mL acetic acid, and 20 mL dichloromethane were stirred overnight at 60 °C. After cooling to room temperature, the solvent was removed, washed by hot ethanol (50 mL) for three times, and then dried by vacuum to afford a red solid (314 mg, yield: 83%). ^{1}H NMR (400 MHz, $CDCl_3$ + TMS, 25 °C): δ 9.46 (d, *J* = 8.4 Hz, 2H), 8.37 (s, 2H), 7.97 (d, *J* = 8.4 Hz, 2H), 7.31-7.27 (m, 8H), 7.21 (d, *J* = 8.7 Hz, 4H), 7.14 (d, *J* = 8.5 Hz, 8H), 7.08-7.04 (m, 8H). HRMS (ESI) m/z calcd for $C_{54}H_{35}Br_2N_6^+$ $[M+H]^+$ 927.1264, found 927.1261.

Device fabrication and characterization

The prepatterned indium tin oxide (ITO) substrates were cleaned by ultrasonic acetone bath, followed by ethanol bath. Afterward, the substrates were dried with N_2 and then loaded into a UV-Ozone chamber. Then the samples were transferred to the deposition system. 1,1-Bis[4-[*N,N*-di(*p*-tolyl)-amino]phenyl]cyclohexane (TAPC, 30 nm) was firstly deposited onto the ITO substrate, consecutively followed by 1,3-carbazolbenzene (mCP, 10 nm), emission layer (20 nm), bis[2-((oxo)diphenylphosphino)phenyl] ether (DPEPO, 10 nm) and

1,3,5-tri(*m*-pyrid-3-yl-phenyl)benzene (TmPyPB, 30 nm). Finally, a cathode composed of Liq (2 nm) and Al (100 nm) was sequentially deposited onto the substrate in the vacuum of 10^{-5} Pa. The voltage-current-luminance characteristics and the EL spectra were simultaneously measured with PR735 SpectraScan Photometer and Keithley 2400 sourcemeter unit under ambient atmosphere at room temperature.

Analyses of rate constants

The rate constants were analyzed according to the literature method [2] with the assumption that $k_{nr,S} << k_{r,S}$, k_{ISC}, i.e. very small non-radiative loss in the singlet state and thus $\Phi_{ISC} \sim (1-\Phi_p)$, particularly for efficient TADF emitters with high PLQY ≥ 90%. Where k_{RISC}, $k_{r,T}$ and $k_{nr,T}$ represent the rate constants of the RISC process, the radiative decay and non-radiative decay from T_1 to S_0 states, respectively. The rate constant of radiative decay from S_1 to S_0 states ($k_{r,S}$), the rate constant of non-radiative decay ($k_{nr,S}$) and k_{RISC} can be obtained:

$$k_p = 1/\tau_p \qquad \text{S1}$$

$$k_d = 1/\tau_d \qquad \text{S2}$$

$$k_{r,S} = \Phi_p k_p + \Phi_d k_d \qquad \text{S3}$$

$$k_{nr,S} = \frac{1-\Phi_{PL}}{\Phi_{PL}} k_{r,S} \qquad \text{S4}$$

$$k_{ISC} \approx k_p(1-\Phi_p) \qquad \text{S5}$$

$$k_{RISC} \approx \frac{k_p k_d \Phi_d}{k_{ISC}\Phi_p} \qquad \text{S6}$$

Φ_p and Φ_d represent quantum yields for the prompt and delayed fluorescence components. With Φ_p, Φ_d, τ_p and τ_d experimentally determined from typical Φ_{PL} and

transient PL data, $k_{r,S}$, $k_{nr,S}$, k_{RISC}, and k_{ISC} can be calculated by Equations S1-6.

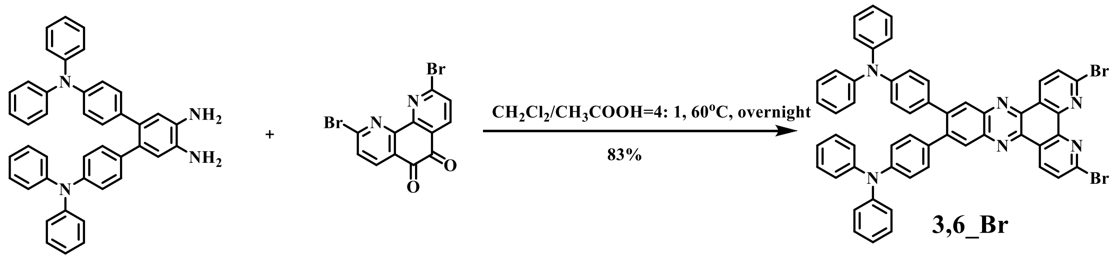

Scheme S1. Synthetic route of 3,6_Br.

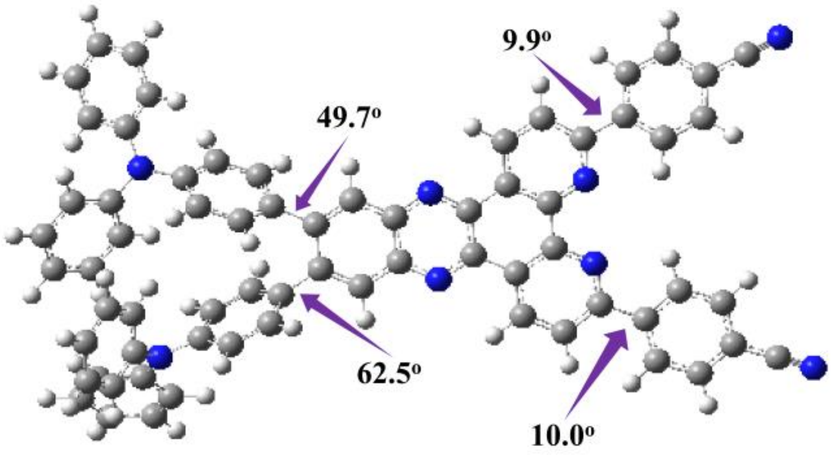

Figure S1. The optimal geometry of the ground state for 3,6_R.

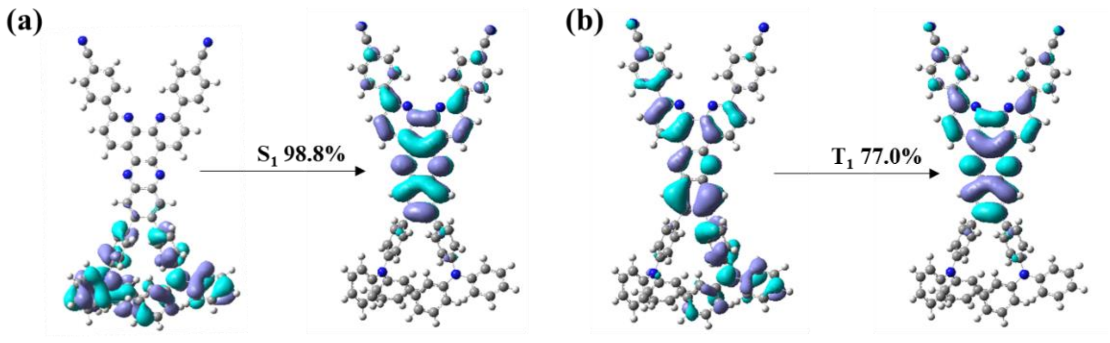

Figure S2. The natural transition orbitals (**NTOs**) of S_1 and T_1 for 3,6_R.

Table S1. Physical properties of 3,6_R.

Compound	HOMO [eV][a)]	LUMO [eV][b)]	E_g [eV][c)]	S_1 [eV][d)]	T_1 [eV][e)]	ΔE_{ST} [eV][f)]	T_d [°C][g)]
3,6_R	-5.32	-3.06	2.26	2.28	2.06	0.22	555

a) Obtained from cyclic voltammograms in CH_2Cl_2 b) Calculated from the equation: $E_{LUMO} = E_{HOMO} + E_g$. c) Obtained from the UV-Vis absorption spectra in CH_2Cl_2 solutions. d) Estimated from the onset of the fluorescence spectra at 300 K. e) Estimated from the onset of the phosphorescence spectra at 77 K. f) The energy gap between singlet and triplet states. g) Decomposition temperature (T_d) with a 5% weight loss.

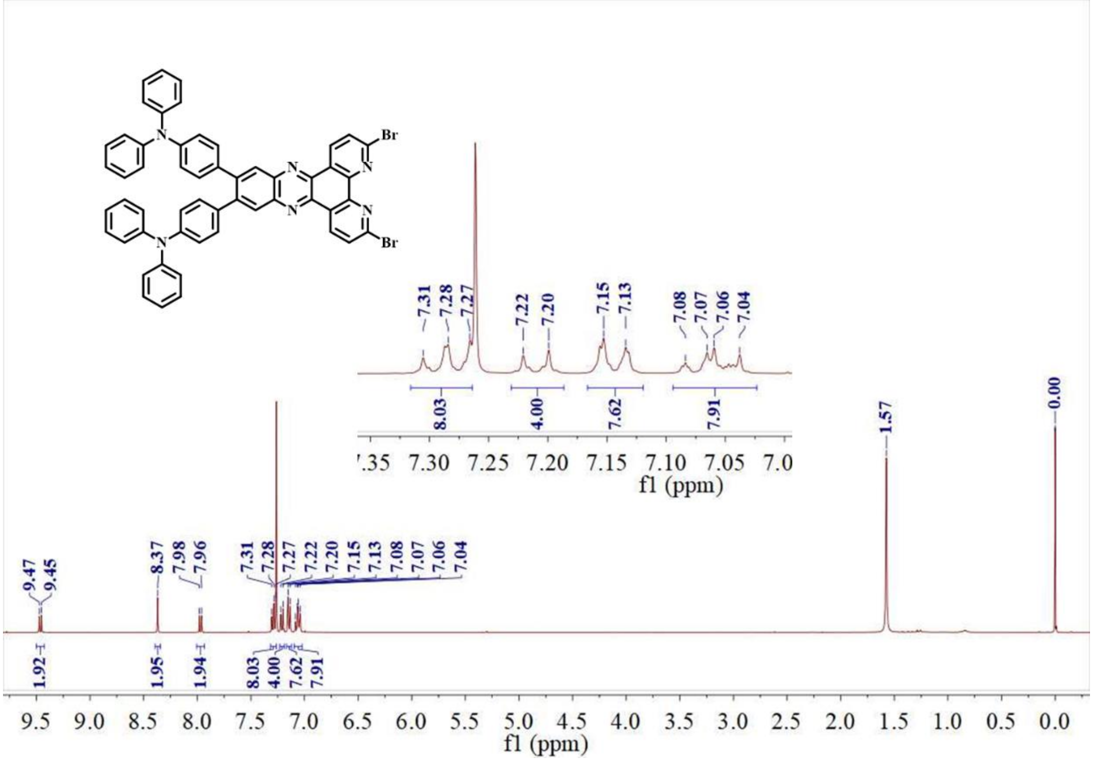

Figure S3. ^{1}H NMR spectra of **3,6_Br** (400 MHz, $CDCl_3$, 25 °C).

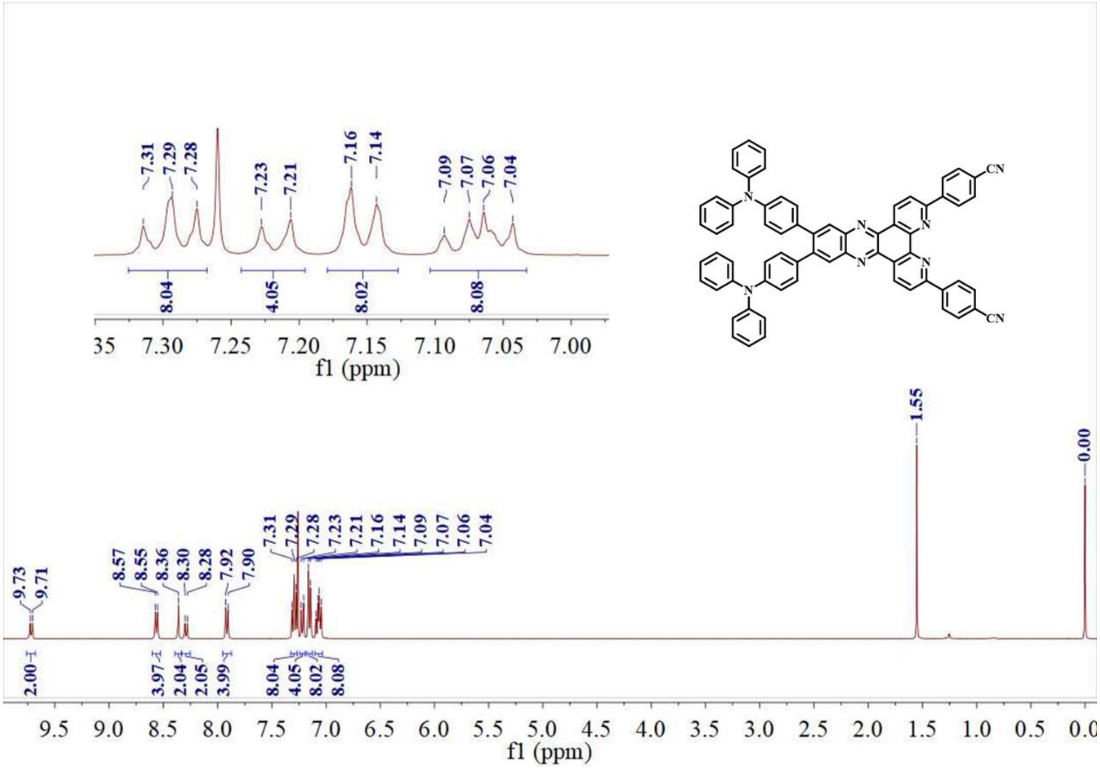

Figure S4. ^{1}H NMR spectra of **3,6_R** (400 MHz, $CDCl_3$, 25 ^oC).

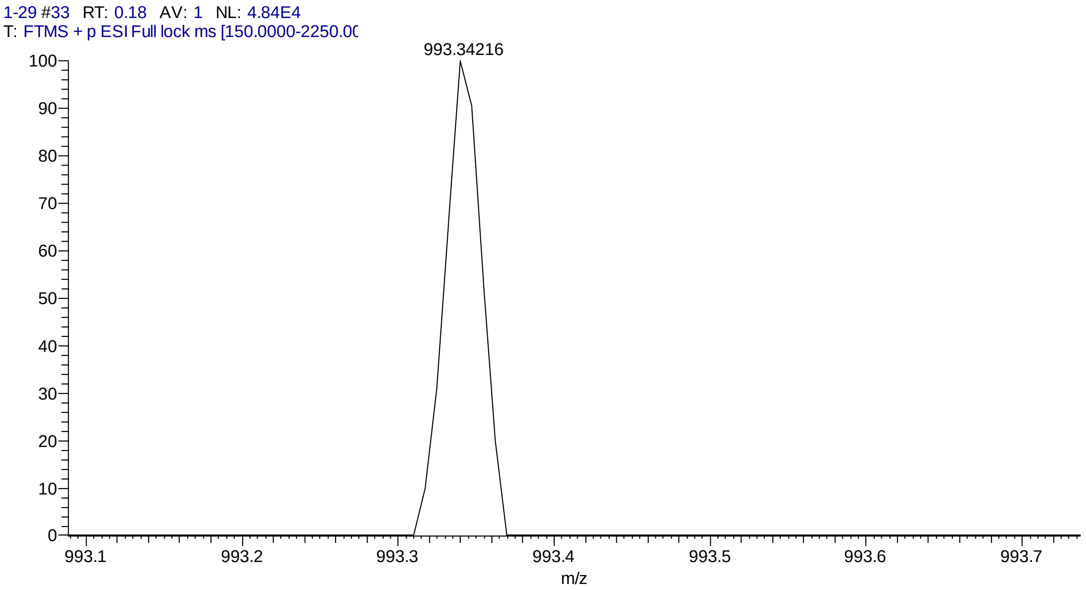

Figure S5. HRMS (ESI) m/z calcd for $C_{68}H_{42}N_8Na^+$ $(M+Na)^+$ 993.3424, found 993.3421.

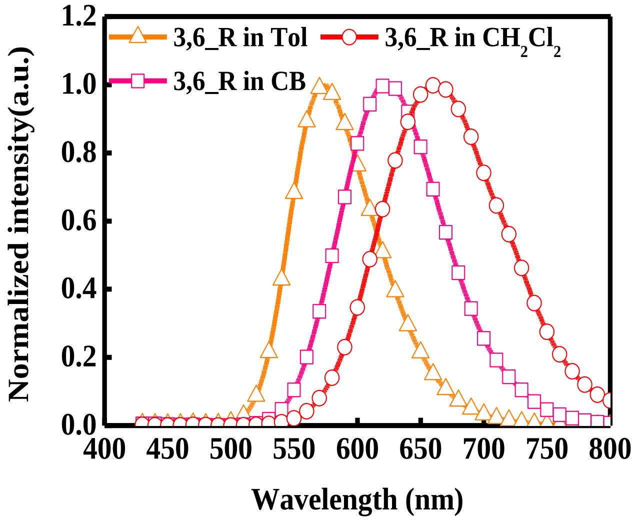

Figure S6. The normalized FL spectra of **3,6_R** in 10^{-5} M toluene (Tol), chlorobenzene (CB), dichloromethane (CH_2Cl_2) solvents, respectively.

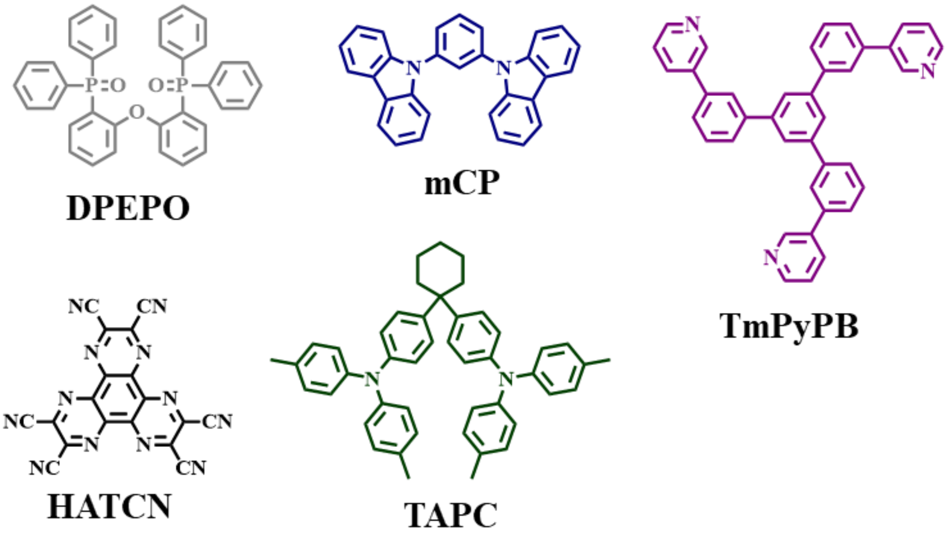

Figure S7. Chemical structures of the materials employed in the vacuum-deposited devices.

Table S2. Summary of rate constants for 10 wt% 3,6_R in the DPEPO host.

Compound	k_p [10^7 s^{-1}]	k_d [10^6 s^{-1}]	$k_{r,S}$ [10^7 s^{-1}]	$k_{nr,S}$ [10^6 s^{-1}]	k_{RISC} [10^5 s^{-1}]	k_{ISC} [10^7 s^{-1}]
3,6_R	5.0	1.0	3.6	5.9	6.7	1.4

Calculated assuming $k_{nr,T}$+ $k_{r,T}$<< k_{RISC}, i.e., most of triplet states can return to singlet states through RISC and thus Φ_{RISC} ≈1 and major nonradiative losses occur in singlet states.

References:

[1] Zhang Y.-L.; Ran Q.; Wang Q.; Liu Y.; Hänisch C.; Reineke S.; Fan J.; Liao L.-S. High-efficiency red organic light-emitting diodes with external quantum efficiency close to 30% based on a novel thermally activated delayed fluore-scence emitter. *Adv. Mater.* **2019**, *31*, 1902368, doi:10.1002/adma.201902368.

[2] Pan K.-C.; Li S.-W.; Ho Y.-Y.; Shiu Y.-J.; Tsai W.-L.; Jiao M.; Lee W.-K.; Wu C.-C.; Chung C.-L.; Chatterjee T.; Li Y.-S.; Wong K.-T.; Hu H.-C.; Chen C.-C.; Lee M.-T. Efficient and tunable thermally activated delayed fluorescence emitters having orientation-adjustable CN-substituted pyridine and pyrimidine acceptor units. *Adv. Funct. Mater.* **2016**, *26*, 7560-7571, doi:10.1002/adfm.201602501.
